# G1-4A, a Polysaccharide from *Tinospora cordifolia* Inhibits the Survival of *Mycobacterium tuberculosis* by Modulating Host Immune Responses in TLR4 Dependent Manner

**DOI:** 10.1371/journal.pone.0154725

**Published:** 2016-05-05

**Authors:** Pramod Kumar Gupta, Pampi Chakraborty, Santosh Kumar, Prafull Kumar Singh, M. G. R. Rajan, Krishna B. Sainis, Savita Kulkarni

**Affiliations:** 1 Radiation Medicine Centre, Bhabha Atomic Research Centre, Mumbai, India; 2 Tuberculosis Aerosol Challenge Facility, International Centre for Genetic Engineering and Biotechnology, New Delhi, India; Public Health Research Institute at RBHS, UNITED STATES

## Abstract

Rapid emergence of drug resistance in *Mycobacterium tuberculosis* (MTB) is a major health concern and demands the development of novel adjunct immunotherapeutic agents capable of modulating the host immune responses in order to control the pathogen. In the present study, we sought to investigate the immunomodulatory effects of G1-4A, a polysaccharide derived from the Indian medicinal plant *Tinospora cordifolia*, in *in-vitro* and aerosol mouse models of MTB infection. G1-4A treatment of MTB infected RAW264.7 macrophages significantly induced the surface expression of MHC-II and CD-86 molecules, secretion of proinflammatory cytokines (TNF-α, IL-β, IL-6, IL-12, IFN-γ) and nitric oxide leading to reduced intracellular survival of both drug sensitive (H37Rv) as well as multi drug resistant strains (Beijing and LAM) of MTB, which was partially attributed to G1-4A induced NO production in TLR4-MyD88 dependent manner. Similarly, bacillary burden was significantly reduced in the lungs of MTB infected BALB/c mice treated with G1-4A, with simultaneous up-regulation of the expression of TNF-α, INF-γ and NOS2 in the mouse lung along with increased levels of Th1 cytokines like IFN-γ, IL-12 and decreased levels of Th2 cytokine like IL-4 in the serum. Furthermore, combination of G1-4A with Isoniazid (INH) exhibited better protection against MTB compared to that due to INH or G1-4A alone, suggesting its potential as adjunct therapy. Our results demonstrate that modulation of host immune responses by G1-4A might improve the therapeutic efficacy of existing anti-tubercular drugs and provide an attractive strategy for the development of alternative therapies to control tuberculosis.

## Introduction

The ability of *Mycobacterium tuberculosis* (MTB) to successfully parasitize the macrophages is a result of its capacity to adapt to the changing host environment by utilizing the resources available to it within host and inhibiting the host responses directed against it [1]. Host processes inhibited by pathogenic species of MTB include fusion of phagosome and lysosome [2, 3], antigen processing [4], responsiveness to IFN-γ [5], production of cytokines, reactive oxygen intermediates, reactive nitrogen intermediates [6] and host cell apoptosis [7–9]. It has also been reported that MTB up-regulates the expression of anti-inflammatory cytokine IL-10 to suppress macrophage activation [10] and host cell apoptosis [8].

Despite the availability of effective treatment of TB, rapid emergence and spread of drug resistant MTB strains, along with need for extended use of current drug regimens, are worsening the burden of disease [11] and thus making it compulsory to explore the novel strategies of anti-tubercular therapies. One such approach involves selective modulation of host immune responses to enhance the therapeutic potential of existing anti-tubercular drugs [12]. This approach is largely used as adjunct therapy to support and enhance the efficacy of antibiotics and antivirals and it has shown promising outcomes in cancer, autoimmune, inflammatory and infectious diseases [12–14]. Innate immune responses in tuberculosis are suboptimal and hence immunomodulation offers the potential to skew the equilibrium back in favor of host either by enhancing or suppressing the selective elements of innate immune system as well as exploiting the strong and intricate effector mechanisms that have evolved over time for pathogen clearance. The discovery of the pathogen recognition receptors of innate immunity, particularly the Toll-like receptors (TLRs), has opened new possibilities for modulation of the innate immune system. Different TLR agonists are being investigated as potential therapeutic agents for the treatment of various diseases, for example, TLR3, 7, 8 and 9 agonists for cancer, TLR4 and 9 agonists for allergies and TLR3, 7 and 9 agonists for viral infections. Further, agonists of TLR 4/5/7/8/9 are being developed as prophylactic and therapeutic vaccines and vaccine adjuvants for the treatment of cancer and viral infectious diseases [15]. CpG DNA, a TLR9 agonist was shown to control the microbial infections by boosting the innate immune responses, indicating antimicrobial potential of such compounds [16].

Crude aqueous extracts of stem of the Indian medicinal plant *Tinospora cordifolia* have been shown to enhance the intracellular bactericidal activity of macrophages and neutrophills in *E*. *coli* induced peritonitis [17, 18]. It was shown to be mitogenic to B cells and its activity based purification resulted in isolation of a polysaccharide, known as G1-4A, an acidic arabinogalactan of m.w. 2.2 x 10^6^ Da [18–20]. Macrophages were the principal target cells of G1-4A whereas it also augmented the DC maturation [18, 19, 21].

Till date, effects of immunomodulators on the intracellular survival of MTB have not been evaluated. Based on earlier reports of G1-4A, we hypothesized that it may act as an immunomodulator to enhance the immune response of the host to clear the intracellular MTB. To prove our hypothesis, in *in vitro* and *in vivo* animal model, we undertook the present study to investigate the effect of G1-4A on intracellular survival of MTB. Our results demonstrate that G1-4A inhibits the survival of drug-sensitive as well as multi-drug resistant (MDR) strains of MTB in *in vitro* and *ex vivo* macrophage model of MTB infection, by inducing nitric oxide (NO) in TLR4-MyD88 dependent manner with simultaneous induction of pro-inflammatory cytokines and surface expression of MHC-II and CD-86. It also reduces the bacterial burden in the lungs of MTB infected BALB/c mice along with induction of Th1 type of immune response.

## Materials and Methods

### Cell line

The murine macrophage cell line RAW 264.7 (American Type Culture Collection) was cultured in DMEM (Gibco Laboratories) supplemented with 10% FBS (Gibco Laboratories) at 37°C in a humidified, 5% CO_2_ atmosphere. Before infection 2.5 X 10^5^ cells per well, were plated in 24 well plates for overnight.

### Bacterial Culture

The MDR strains of *M*. *tuberculosis* strain-1 (Beijing), and strain-2 (LAM); were isolated from patients in Tata memorial hospital and KEM hospital, Mumbai (INDIA), and characterized as described earlier [22]. The laboratory strain H37Rv was also included in the study. *M*. *tuberculosis* isolates were grown in Middlebrook 7H9 medium (Difco) supplemented with 10% ADC (Becton Dickinson) and 0.05% Tween 80 (Sigma) at 37°C with daily agitation until the mid log phase. Working stocks were prepared (10^8^ bacilli/ml) in DMEM and stored at -80°C until use. All procedures were carried out in a Bio safety Level III (BSL III) laboratory.

### Animals

Six-week old female BALB/c mice (National institute of Nutrition, Hyderabad, India), free of common pathogens, were used for all experiments. Mice were housed under specific-pathogen-free conditions in bio safety level III facilities at the Tuberculosis Aerosol Challenge Facility (International Centre for Genetic Engineering and Biotechnology, New Delhi, India). Experimental groups of BALB/c mice were matched for age (within 1 to 2 weeks) and gender both, for each experiment. During entire study, no infected animals died (without euthanasia) before reaching the experimental endpoints (ie. before 15, 30 or 60 days post infection). Condition of animals was monitored every day by veterinary in charge of the facility. No extra steps were taken to minimize suffering of the animals, including analgesics and anaesthetics.

### Ethics statement

Bhabha Atomic Research Centre Animal Ethics Committee (BAEC) approved the present study (Project no-BAEC/16/11). Standard operating protocols approved and created by Tuberculosis Aerosol Facility at International Centre of Genetic Engineering and Biotechnology, New Delhi (ICGEB-TACF) were strictly followed.

### Extraction of polysaccharide G1-4A from *T*. *Cordifolia*

G1-4A was isolated and purified from *Tinospora cordifolia*, an Indian medicinal plant, as described in the Indian Patent no. 56/Bom/98 [20]. Briefly, methanol was added to powdered dry stems of *T*. *Cordifolia* to remove low molecular weight constituents, followed by acetone precipitation to obtain polysaccharide rich fraction. Protein impurities were removed by trichloroacetic acid (TCA) precipitation followed by column chromatography on a Sephacryl S-400 gel for further purification. The main constituents of the polysaccharide as estimated by GC were galactose (32%), galacturonic acid (35%), arabinose (31%), and rhamnose (1.4%). GC–MS analysis predicted the presence of 1,6-linked galactose, terminal galactose, 1,4-linked galactose, terminal arabinose, and 1,5-linked arabinose [20]. Chintalwar et al characterized G1-4A, an acidic arabinogalactan polysaccharide on the basis of these observations [20]. The G1-4A used in the present study was devoid of endotoxin contamination as estimated by the limulus amebocyte lysate (LAL) assay using the E-TOXATE kit. The biological activity of the purified fraction was determined by its TNF-α producing properties in RAW 264.7 cells and primary murine macrophages.

### Measurement of cytokines

The levels of cytokines such as TNF-α, IL-12, IL-6, IL-4, IL-10, IL-1β and IFN-γ the culture supernatants of macrophages infected with MTB strains or serum of infected animals were measured using BD OptEIA mouse ELISA sets (BD Biosciences) according to the manufacturer’s instructions. Briefly, monoclonal capture Ab were coated in 96 well ELISA plates for overnight at 4°C, followed by addition of 100 μl of supernatant in each well and incubated for 2 h at room temperature. Subsequently biotinylated monoclonal detection Ab and streptavidin POD were added for 1 h at RT followed by addition of chromogenic substrate (3,3',5,5'-Tetramethylbenzidine, TMB) to give a colored product. Stop solution was added and absorbance was measured at 450 nm. Subsequently concentrations of cytokines in samples were extrapolated from standard curve.

### Nitric oxide assay

RAW 264.7 cells were treated with G1-4A for 8h prior to infection with MTB strains and followed by treatment with G1-4A for 48 h. The concentration of nitrite, which is the stable end product of NO, was determined by Griess’ reaction in the supernatants [23]. Sodium nitrite standard curve was used to extrapolate the Nitrite content in each experiment.

### Western-blot analysis

Cells were harvested, washed thrice with cold PBS followed by lysis in REPA lysis buffer containing 1% protease inhibitor cocktail (Sigma) and 3% phosphatise inhibitor cocktail (Sigma). Total protein concentration in cell lysate was evaluated using Bradford reagent (Sigma) and lysates containing equal amounts of protein (50 μg/well) were resolved by SDS PAGE and electroblotted to nitrocellulose membrane. Membranes were blocked in 5% w/v skimmed milk in 1X TBST for 30 minutes and incubated with rabbit polyclonal anti-TLR4 (1:1000 v/v), anti-MyD88 (1:1000 v/v), and anti-NOS2 (1:1000 v/v, all from Santa Cruz Biotechnology). Rabbit polyclonal anti–β-actin (1:500 v/v, Cell Signalling Technology), was used as loading control. Membranes were washed with 1xTBST and incubated with Goat anti-rabbit secondary antibody HRP conjugate (1:2000 v/v, polyclonal, Roche) for 2 h and visualized with Enhanced Chemiluminisence kit (Roche) according to manufacturer’s protocol.

### Flow cytometry

Macrophages (5 X 10^5^ cells/well in 24-well plates) either uninfected or infected with MTB strains were incubated in the presence or absence of G1-4A for 24 h and 48 h. Macrophages were harvested, washed thrice with 1XPBS and incubated with CD16/CD32 Fc block antibodies (Biolegend) to block FcRs. In next step cells were stained with following fluorochrome-conjugated primary antibodies: FITC rat anti-mouse CD86 (clone PO3), FITC rat anti-mouse (IA^d^) MHC-II (clone 39-10-8, all from Biolegend). FITC labeled appropriate isotype control antibody (Biolegend) were used as labeling controls. Cells were washed and fixed with 4% para formaldehyde and analyzed with CyFlow^®^ Space flowcytometer (Partec). Cells were gated according to their forward versus side scatter. Positively stained cells were gated using appropriate FITC labelled isotype antibodies as negative controls using FlowJo software (Treestar).

### siRNA transfection and blocking of TLR4 by anti-TLR4 blocking antibodies

To study the involvement of TLR4 and MyD88 pathway we used siRNAs against TLR4 and MyD88 or anti-TLR4 blocking antibodies. Cells were transfected with siRNA at a final concentration of 100 nM using the Xtreme GENE transfection reagent (Roche) according to manufacturer’s protocol. G1-4A treatment was given 48 h after transfection. In other experiments cells were incubated with anti-TLR4 blocking antibody for 1 h prior to the treatment with G1-4A. During infection studies cells were first infected with MTB, followed by transfection with siRNAs or treatment with blocking antibody.

### Phagocytosis Assays

RAW 264.7 cells were grown on cover slips in six well plates, and either treated with G1-4A, LPS or left untreated 8 h prior to the infection with GFP expressing MTB at MOI 10. Cells were incubated with GFP-MTB H37Rv at 37°C in a 5% CO2 environment for two hours. After infection, macrophages were washed thrice with 1X PBS and treated with 10μg/ml amikacin to get rid of extracellular bacteria. Cells were again washed with 1X PBS, fixed with 4% para formaldehyde. DAPI was used as counter stain. Cover slips were mounted onto slides with Prolong Gold Antifade (Invitrogen) and analyzed on a Nikon TE 2000E laser scanning confocal microscope. For evaluation of phagocytosis 300 macrophages per well were counted at 63X magnification under oil immersion and serial optical sections with Z-stack spanning were taken to monitor colocalization and images were deconvolved. Cells containing at least one ingested bacterium were counted to estimate the percent of infected macrophages, and the mean number of internalized bacteria was multiplied to generate phagocytic index. Trypan blue (final concentration, 0.025%) was used to quench extracellular bacterial fluorescence.

Phagocytic index=(percentage of macrophages containing at least one bacterium)X(mean number of bacteria per positive cell).

In another set of experiments cells either pre treated with G1-4A for 8 h or untreated, were infected with GFP-MTB for 4 h. Cells were harvested and their intracellular fluorescence was analyzed by flow cytometer (Partec).

### Macrophage infection and CFU assay

MTB H37Rv, clinical isolates Beijing and LAM, growing in mid-log growth phase were centrifuged; pellets were washed with PBS (pH 7.4) and resuspended in DMEM medium. Cells were vortexed with the glass beads in 20 ml glass tube, followed by passage through 26G needle for 8–10 times to remove cell clumps and prepare single cell suspension. The dispersed bacteria were allowed to stand for 15 min and the upper half of the bacterial suspension was then used for the experiments. RAW 264.7 cells were seeded in 24 well tissue culture plates at a density of 2x10^5^ cells/well and treated with G1-4A, 8–12 h prior to infection with MTB strains at an MOI of 5. Four hours post infection, cells were washed thrice with pre warmed media and incubated with medium containing 10 μg/ml of amikacin for 1 h to kill extracellular bacteria and again treated with G1-4A (1 mg/ml) for 24, 48 and 72 h. Supernatants were stored at -80°C at each time points for estimation of cytokines and NO levels. For CFU assay, infected cells were washed thrice with sterile PBS and lysed with 0.01% SDS in PBS at different time points. Serial dilutions were prepared in PBS and plated on Middlebrook 7H11 medium supplemented with OADC and colonies were counted after 3 weeks.

### Aerosol Infection and drug treatment

For each experiment, frozen stocks of bacteria were thawed and passed through 26G needle to disperse the clumps. Aerosol exposure chamber (U.V. Madison College of Engineering Shops, Cambridge Square Inc.) was used to generate the aerosol. For each experiment BALB/c mice (6 per group), either injected with G1-4A (12.5 mg/kg of body weight) through tail vein twice with an interval of 48 h or untreated; were exposed to MTB strains in the aerosol chamber for 15 min with an estimated aerosol dose of 150–200 bacilli per animal, as determined by the culture of lung homogenates of one mouse from each group, after 24 hours. Seven days post infection, G1-4A (12.5 mg/kg of body weight) was injected into the tail vein of mice again. G1-4A treatment was repeated after every 72 hours till 15, 30 or 60 days. Mice were sacrificed by carbon dioxide narcosis after 15, 30 and 60 days of infection. The lungs were removed aseptically and weighed. The lungs were then homogenized and appropriate dilutions of lung homogenates were plated on Middlebrook 7H11 agar plates for subsequent enumeration of the CFU after 3 weeks.

### Reverse Transcription (RT)-PCR for Cytokine mRNA Detection

The lower lobes of the left lung from three mice per group were used to isolate total RNA using RNeasy Mini Kit (QIAGEN) according to manufacturer’s protocol. cDNA was prepared from total RNA using RevertAid M-MuLV Reverse Transcriptase (Fermentas). Real-time PCR was performed using the Mx3000P real-time PCR system (Stratagene) and SYBR Green Mastermix kit (Agilent). The cDNA was amplified using specific primers (S1 Table) deduced from the published sequence [24]. GAPDH was used as internal control. The thermal cycling profile for PCR amplifications were as follows: denaturation for 30 s at 94°C, annealing for 30 s at 58°C and then extension for 1 min at 72°C X 40 cycles) followed by melt curve analysis. Relative quantitation of gene expression was performed using 2^-ΔΔCt^ method [25].

### *In vitro* restimulation Assay

Spleen cells from mice were dissociated by squeezing the spleen through a sterile nylon mesh in a petri plate containing RPMI medium. Red blood cells were lysed by treatment with 0.83% ammonium chloride for 5 min. Splenocytes were washed thrice with 1X sterile PBS and resuspended in RPMI. Splenocytes (2.5x10^6^ cells/ml) were incubated either in 2 ml of complete media or medium containing 5 μg/ml concanavalin A (ConA), or PPD of MTB (3 μg/ml) or medium alone for 72 h at 37°C. Levels of IFN-γ and IL-4 were assayed in culture supernatants by ELISA as per manufacturer’s protocol (BD Biosciences). To ensure specifically that IFN-γ and IL-4 estimated were produced by T helper cells, splenocytes were divided in two groups, and one group was incubated with anti MHC-II (IA^d^) antibodies for 1 h prior to the treatment with ConA or PPD, while other group was used as control.

### Statistical analysis

Data obtained from independent experiments were presented as mean ±SD and analyzed by either paired Student’s t-test or one way analysis of variance (for multiple comparisons) by using Sigma Stat software (version 3.5). Differences were considered statistically significant at p value <0.05.

## Results

### G1-4A modulates the host protective immune responses in macrophages infected with drug-sensitive and MDR strains of MTB

**Fig 1:**
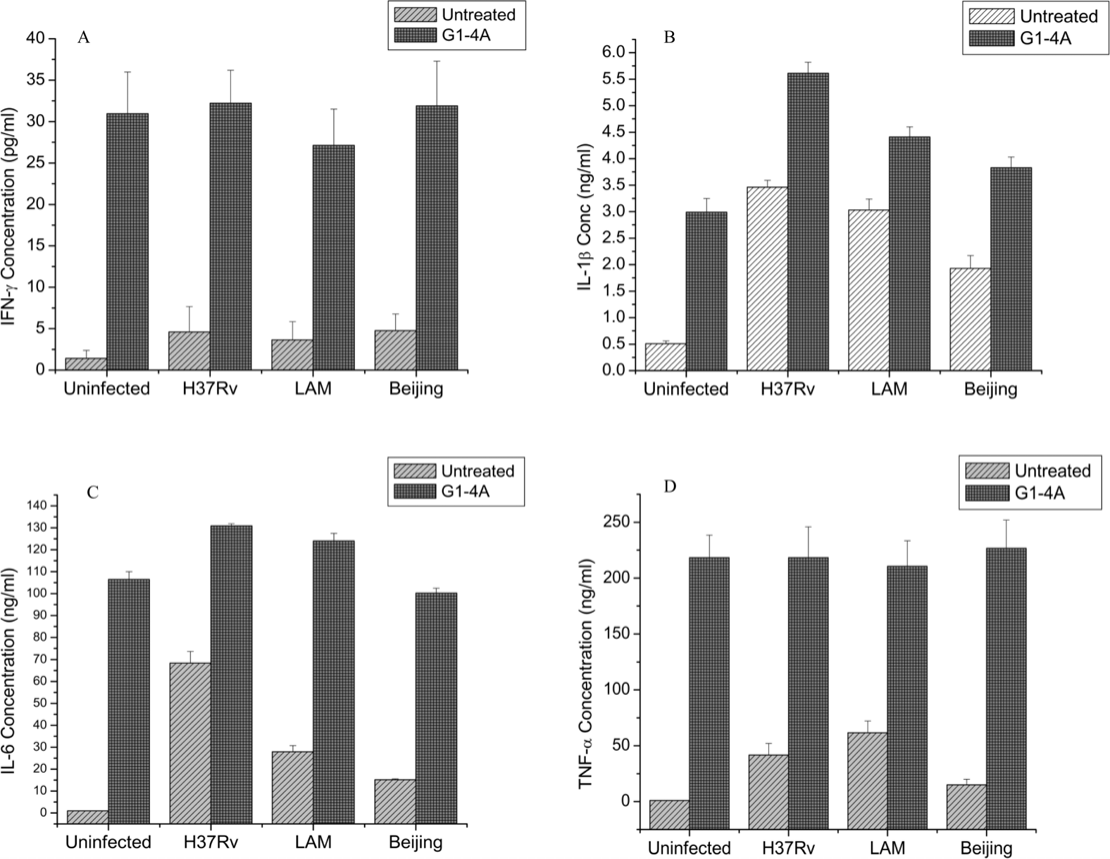
G1-4A mediated cytokine production by MTB infected RAW cells. RAW cells were treated with G1-4A (1 mg/ml), for 8 h and infected with three strains of MTB i.e. H37Rv, LAM and Beijing at MOI 5. Four hours after infection cells were retreated with G1-4A for 24 h. Cytokines levels were quantified in supernatants by ELISA, (A)- IFN-γ, (B)-IL-1β, (C)-IL-6, (D)-TNF-α. Results shown here are from a single representative experiment out of at least three independent experiments performed and presented as mean ± SD.

To determine the effect of purified G1-4A on the expression of pro-inflammatory cytokines in MTB infected macrophages, murine macrophage cell line RAW 264.7 cells were incubated with G1-4A for 8 h followed by infection with MTB H37Rv, a drug-sensitive laboratory strain and two MDR clinical isolates of MTB belonging to two different genetic lineages viz: Beijing and LAM. The cells were subsequently treated again with G1-4A. The effects of G1-4A on cytokines in MTB infected RAW cells were then analyzed in the culture supernatants. MTB H37Rv, Beijing and LAM differentially regulated the expression of cytokines in macrophages. Our cytokine data demonstrated that IFN-γ levels were very low in supernatants of cells infected with all the MTB strains in absence of G1-4A but increased significantly after treatment with G1-4A (Fig 1A). In case of IL-1β, differential expression was observed in untreated cells, with H37Rv infected cells showing the highest and Beijing showing the lowest levels (Fig 1B). However, G1-4A treatment up-regulated the levels of IL-1β in cells infected with all the strains. Similar pattern was also observed in case of IL-6 (Fig 1C). In case of TNF-α, LAM induced maximum and Beijing induced minimum level, although G1-4A treatment yet again increased the production of TNF-α in cells infected with all the strains (Fig 1D). Therefore, it was evident that G1-4A modulated the expression of pro-inflammatory cytokines in MTB infected cells irrespective of the genotype, lineage and drug resistance status of MTB strains.

**Fig 2:**
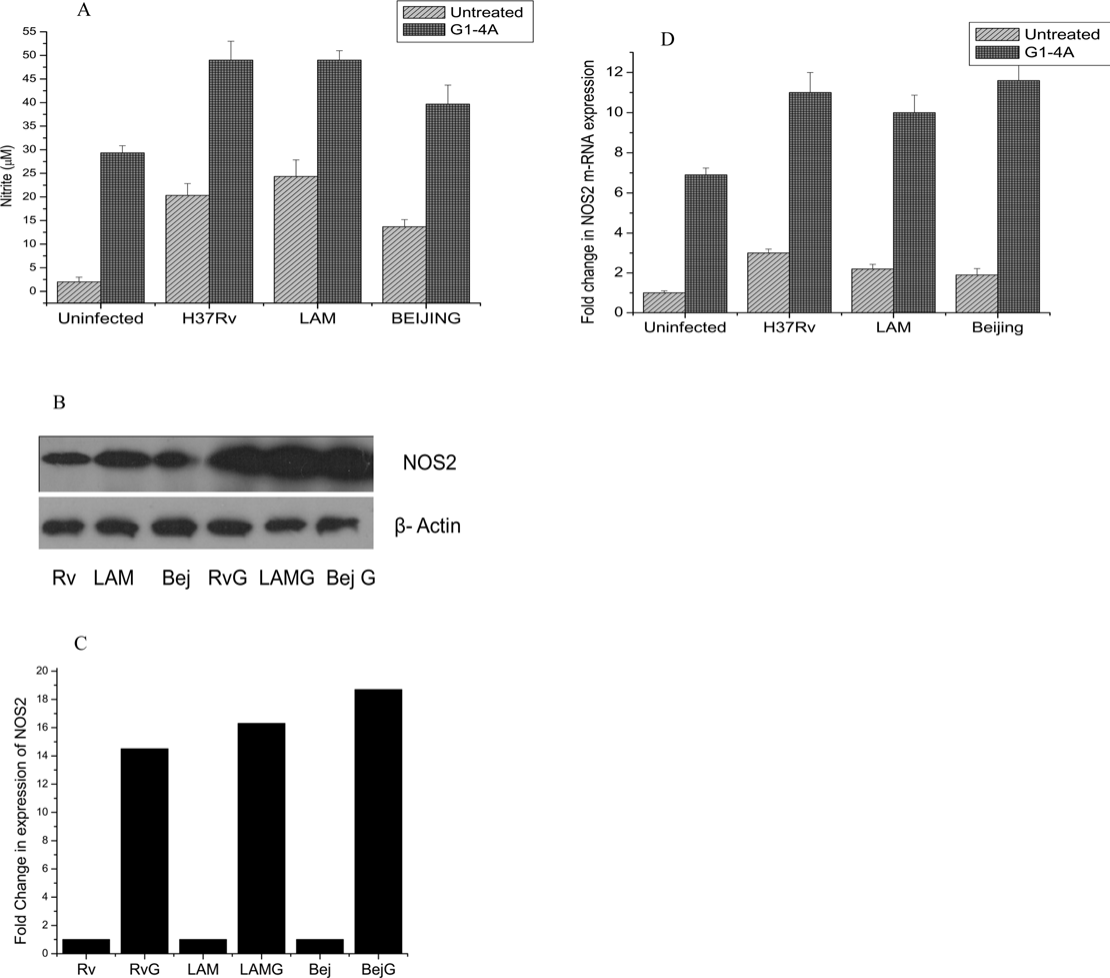
G1-4A mediated nitric oxide production by MTB infected RAW cells. RAW cells were treated with G1-4A and infected with three strains of MTB as described earlier. (A) Nitric oxide levels were measured as nitrite concentration in supernatants by using Griess’ Reagent. (B) Immunoblotting using anti-NOS2 antibodies to determine the protein level of NOS2. (**RvG**- cells infected with MTB H37Rv and treated with G1-4A, **LAMG**- cells infected with MTB LAM and treated with G1-4A, **BejG**- cells infected with MTB Beijing and treated with G1-4A) (C) Densitometric analysis of NOS2 protein expression by Image J (D) Relative quantification of NOS2 mRNA was performed by 2^-ΔΔCt^ method to determine the fold change in NOS2 gene expression compared to GAPDH. Results shown are representative of at least three independent experiments and presented here as mean ± SD.

Further, we investigated the effect of G1-4A on nitric oxide production in MTB infected cells and observed that Beijing induced minimum while LAM induced maximum levels of NO in the absence of G1-4A, but strikingly, levels of NO increased significantly after G1-4A treatment irrespective of MTB lineage or drug resistance status (Fig 2A). Additionally, immunoblotting and real time RT PCR data also suggested G1-4A mediated up regulation of NOS2 at protein and mRNA level in MTB infected cells (Fig 2B–2D)

**Fig 3:**
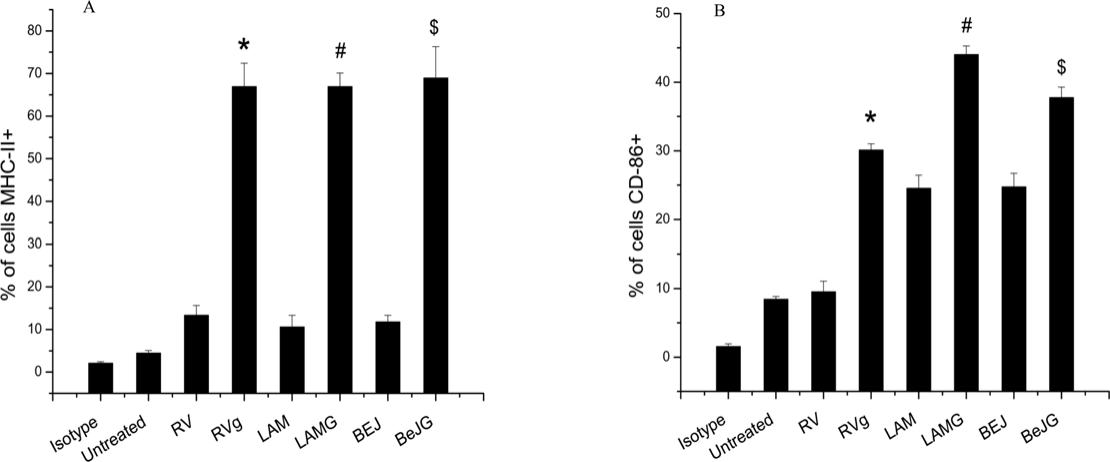
Effect of G1-4A on CD-86 and MHC-II expression in MTB infected RAW cells. RAW cells were treated with G1-4A and infected with three strains of MTB. Post G1-4A treatment cells were incubated with FITC I-A^d^, FITC anti–CD86 and FITC IgG2a and surface expression was evaluated by flow cytometry. (A) and (B) are quantitative representation of percentage of cells MHC-II+ and CD-86+ (*p<0.01 as compared to untreated H37Rv infected control, #p<0.01 compared to untreated LAM infected control and $p<0.01 compared to Beijing infected control, values of at least three independent experiments presented as Mean ± SD).

We also monitored the effect of G1-4A on surface expression of MHC-II and CD86 in MTB infected cells by immunophenotyping. A significant increase in percentage of MHC-II positive cells from 13.33±2.31 percent to 66.93±5.49 percent in case of H37Rv (p<0.01), from 10.59±2.72 percent to 66.1±3.16 percent in case of LAM (p<0.01) and from 11.77±1.57 percent to 68.9±7.35 percent in case of Beijing (p<0.01) infected cells after 24 h of G1-4A treatment was observed (Fig 3A). Likewise, G1-4A treatment augmented the percentage of CD86 positive cells from 9.50±1.56 percent to 30.1±0.92 percent in case of H37Rv (p<0.01), from 24.53±1.91 percent to 44.0±1.25 percent in case of LAM (p<0.01) and from 37.73±1.53 percent to 37.7±1.55 percent in case of Beijing (p<0.01) infected cells (Fig 3B).

Above observations indicated the activation of macrophages infected with MTB by G1-4A treatment which may trigger Th1 responses *in vivo* and enhance the protective immunity.

### G1-4A treatment augments the phagocytosis of MTB in murine macrophages

**Fig 4:**
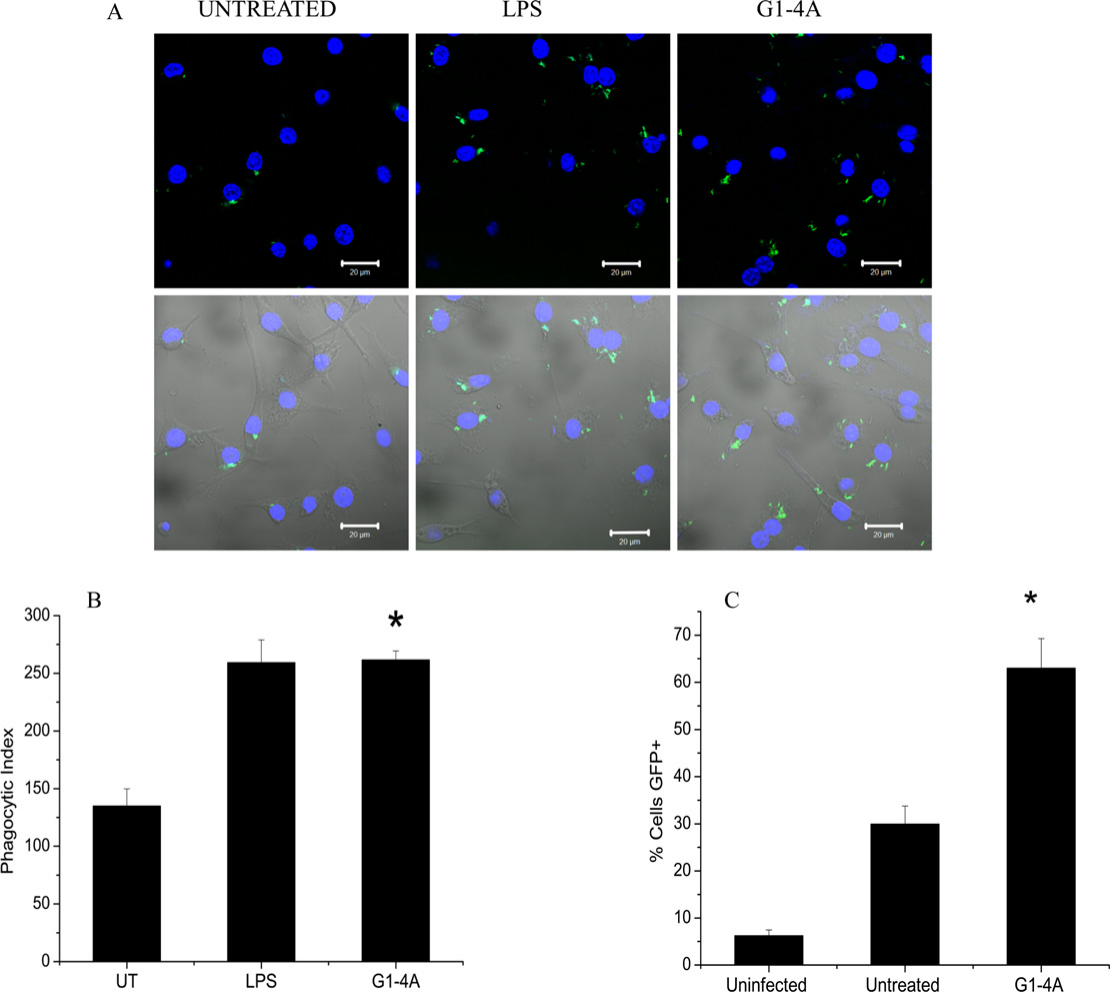
Effect of G1-4A on phagocytic uptake of MTB by RAW cells. (A) Confocal microscopy images of RAW cells infected with GFP tagged MTB H37Rv either treated with G1-4A/LPS or left untreated (DAPI counter stain) (B) Phagocytic index (*p<0.01 compared to untreated control) (C) Quantitative representation of percentage of GFP+ cells as analyzed by Flow cytometer. Values of at least three independent experiments presented as Mean ± SD).

Activated macrophages are known to have enhanced phagocytic ability; therefore, we evaluated the effect of G1-4A on phagocytosis of GFP tagged MTB. RAW cells seeded on cover slips were treated with G1-4A or LPS and infected with GFP-H37Rv. Subsequently cells were fixed using 4% para formalin and visualized under Nikon TE 2000E laser scanning confocal microscope. It was observed that phagocytic uptake of GFP-MTB was significantly higher in cells treated with G1-4A or LPS compared to untreated cells (Fig 4A). Phagocytic indices of G1-4A and LPS treated cells were comparable and were significantly higher as compared to that of untreated control (p<0.05, Fig 4B). Flow cytometry data demonstrated that percentage of GFP positive cells increased significantly, 56.4±6.6 percent, (p<0.01) in G1-4A treated cells, compared to untreated, 30±3.8 percent or uninfected control (background control), 6.2±1.2 percent (Fig 4C).

### G1-4A inhibits the intracellular survival of drug-sensitive and MDR strains of MTB in macrophages partially through nitric oxide production in TLR4-MyD88 dependent manner

**Fig 5:**
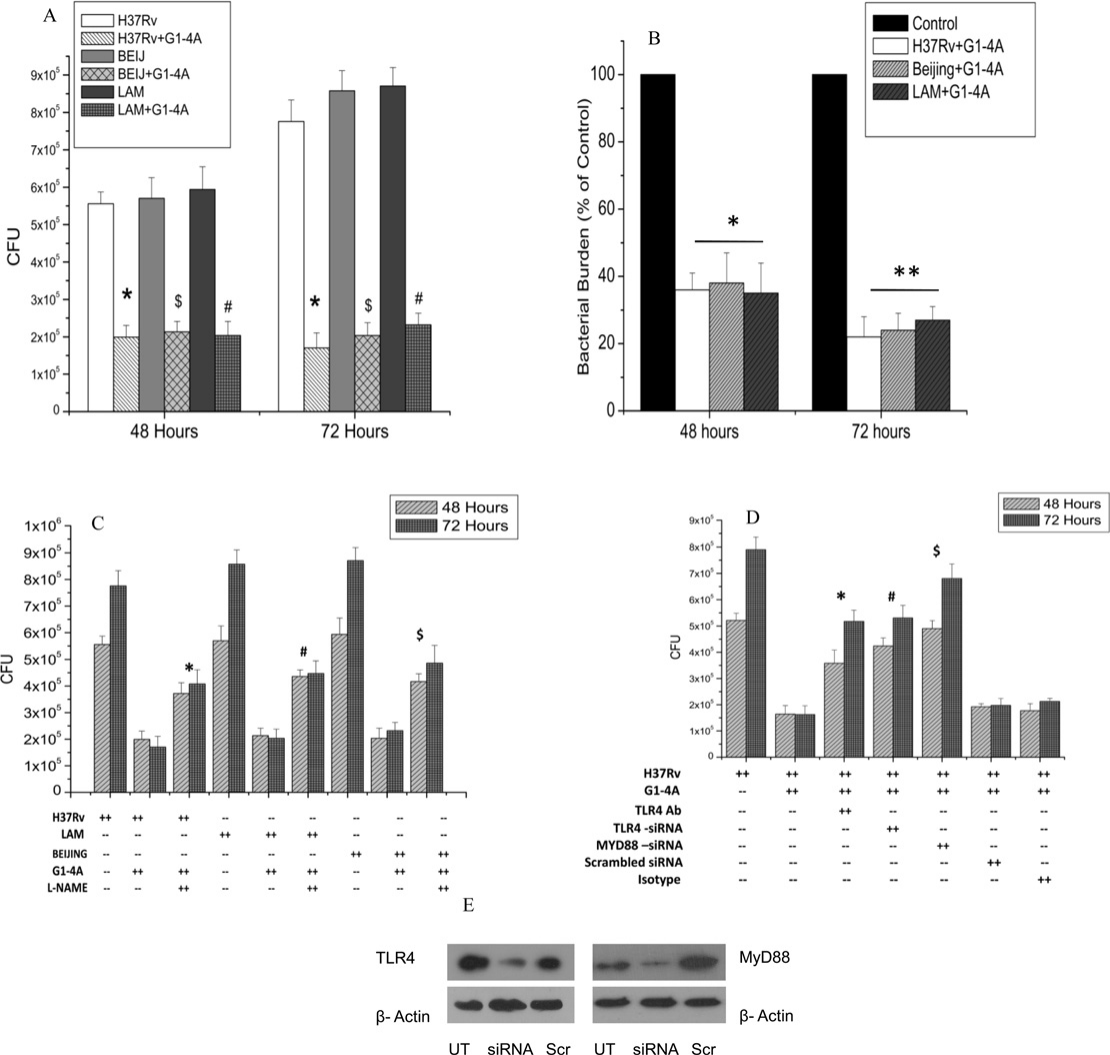
Inhibition of intracellular survival of MTB strains in macrophages by G1-4A. (A) CFU counts of three strains of MTB in presence or absence of G1-4A (*p<0.01 as compared to untreated H37Rv infected control, #p<0.01 compared to untreated LAM infected control and $p<0.01 compared to untreated Beijing infected control). (B) Bacterial burden (Percentage of control, *p<0.05, **p<0.01). (C) CFU assay of RAW cells infected with MTB strains and treated with G1-4A in the presence or absence pharmacological inhibitor of NOS2, L-NAME (100 μM), and incubated for 48 and 72 h. (D) Cells were infected with MTB H37Rv and incubated in the presence of anti TLR-4 blocking antibodies or isotype control antibodies for 2 h followed by treatment with G1-4A for 48 and 72h. In other set of experiments, expression of TLR4 and MyD88 was transiently knocked down by adding siRNA against TLR4 and MyD88. Scrambled siRNA was used as control. Later, cells were infected with H37Rv and treated with G1-4A for 48 and 72 h. Figure depicts CFU data for both the sets of experiments. (E) Immunoblotting of TLR4 and MyD88 in RAW cells after siRNA treatment. Results shown are representative of at least three independent experiments and presented here as mean ± SD, *p<0.05 as compared to only G1-4A treated H37Rv infected control, #p<0.05 compared to only G1-4A treated LAM infected control and $p<0.05 compared to only G1-4A treated Beijing infected control).

Since G1-4A was able to augment the expression of proinflammatory cytokines and skew the host immune responses towards host protection we decided to investigate its effect on the survival of intracellular MTB. RAW cells were treated with G1-4A for 8 h prior to infection with MTB H37Rv, LAM and Beijing strains. After removal of non-internalized bacteria, G1-4A was added for 48, and 72 h and intracellular MTB survival was monitored by CFU enumeration. Colony counts were significantly decreased (Fig 5A) in case of MTB H37Rv (p<0.05), LAM (p<0.05) and Beijing (p<0.05) and a marked decrease in percent bacterial burden (Fig 5B) was observed i.e. 64±5 percent in case of H37Rv (p<0.05), 62±9 percent in case of LAM (p<0.05) and 65±9 percent in case of Beijing (p<0.05) after 48 h of G1-4A treatment and 78±6 percent in case of H37Rv, 76±5 percent in case of LAM and 73±4 percent in case of Beijing after 72 h of G1-4A treatment as compared to untreated MTB infected cells. REMA assay was used to determine the direct anti-mycobacterial property of G1-4A and results of this assay confirmed that G1-4A did not target MTB directly (S1 Fig) but its anti-mycobacterial property is result of host immunomodulatory activity.

As nitric oxide produced by activated macrophages is known to confer bactericidal activity on macrophages, we used L-NAME, a pharmacological inhibitor of NO to determine the role of NO in G1-4A mediated inhibition of intracellular survival of MTB strains. Treatment of L-NAME partially abrogated the G1-4A mediated intracellular inhibition of MTB (Fig 5C) and led to increase in the CFU counts, though counts were comparably lower than control, which suggested that G1-4A induced NO, was partially responsible for G1-4A mediated inhibition of intracellular survival of MTB strains. We had already established that G1-4A induced NO in RAW cells by up regulating NOS2 expression in TLR4-MyD88 dependent manner (unpublished data) therefore, to determine the involvement of TLR4-MyD88 pathway in G1-4A mediated intracellular inhibition of MTB, RAW cells were first infected with MTB H37Rv followed by transfection with siRNA against TLR4, MyD88 and scrambled siRNA to avoid the interference in MTB uptake by siRNAs. After treatment of G1-4A for 48 and 72 h, cells were lysed, diluted and plated on Middlebrook 7H11 plates for CFU enumeration. In presence of TLR4 and MyD88 siRNA, CFU counts were increased compared to CFU in presence of G1-4A alone or scrambled siRNA (Fig 5D). This suggested a role of TLR4 and MyD88 in G1-4A mediated intracellular inhibition. It was further confirmed by addition of anti-TLR4 blocking antibodies in MTB infected RAW cells prior to G1-4A treatment. As shown in Fig 5D, CFU counts increased in presence of anti-TLR4 blocking antibody compared to isotype antibody control or G1-4A alone, corroborating the involvement of TLR4 in G1-4A mediated intracellular inhibition of MTB growth. Additionally, decrease in the expression of TLR4 and MyD88 in RAW cells after siRNA treatment was shown by immunoblotting (Fig 5E). Hence, collectively all the results obtained so far, conclude that G1-4A inhibits the intracellular survival in macrophages partially through the induction of NO in TLR4-MyD88 dependent manner.

### G1-4A inhibits the survival of MTB in mice lungs

**Fig 6:**
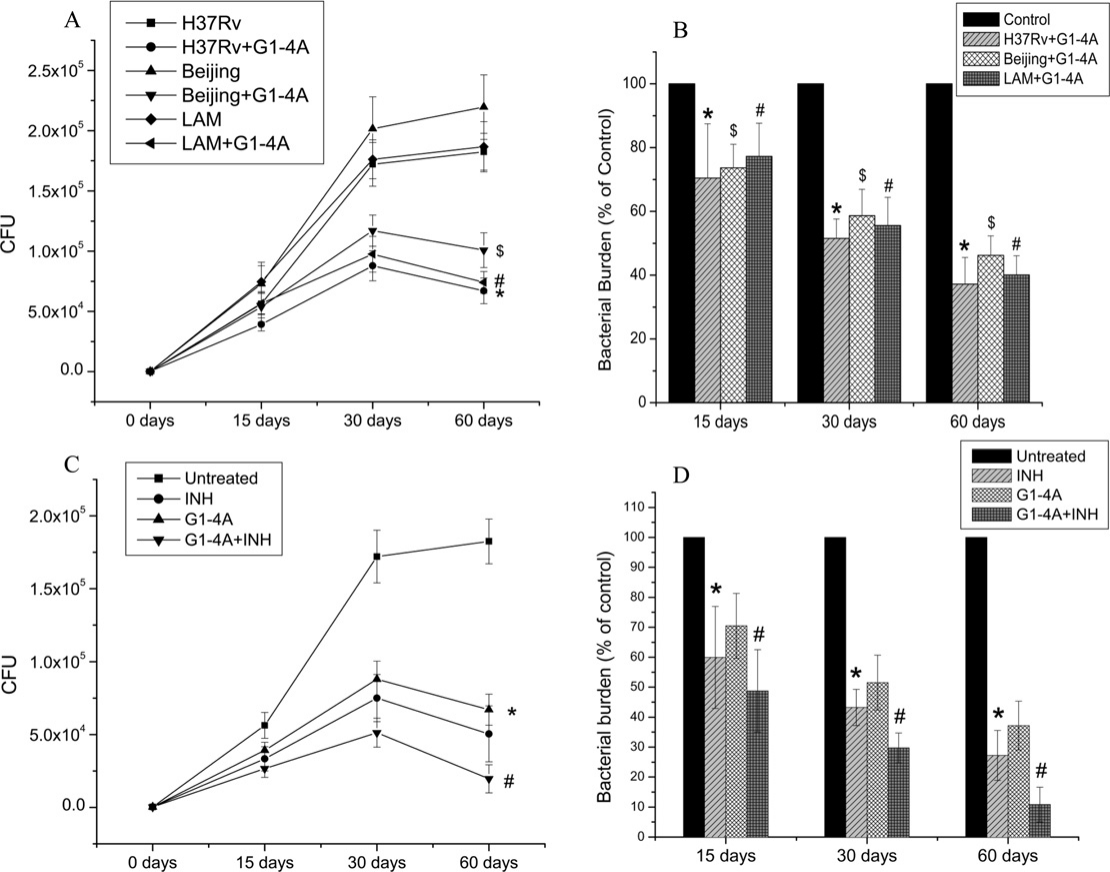
Effect of G1-4A on bacterial burden in MTB infected mice. BALB/c mice infected with MTB strains H37Rv, LAM and Beijing in the presence or absence of G1-4A were sacrificed at 15, 30 and 60 days post infection. Lungs were homogenized and plated on to Middlebrook 7H11 plates after appropriate dilutions. (A) CFU counts (B) Bacterial burden as percentage of untreated controls were plotted, *p<0.05, $p<, 0.05 #p<0.05 compared to untreated. (C) In another set of experiment mice were infected only with H37Rv and treated with G1-4A, INH alone or in combination. Animals were sacrificed and lung homogenates were plated onto Middlebrook 7H11 plates for CFU enumeration (D) Bacterial burden as percentage of untreated controls *p<0.05, $p<, 0.05 #p<0.01 compared to untreated. Results shown here are representative of at least three independent experiments performed and presented as mean ± SD.

Inhibition of intracellular survival of MTB strains in macrophages by G1-4A was attributed to activation of macrophages which enhanced their antimicrobial properties. To further validate the significance of targeting host, we decided to investigate the anti-mycobacterial efficacy of G1-4A in mouse infection model. BALB/c mice were pretreated (i.v.) with G1-4A, 96 h and 48 h prior to aerosol infection with MTB strains H37Rv, LAM and Beijing. Seven days post infection G1-4A treatment was started again and given after each 72 h till 15, 30 and 60 days post infection. Animals were sacrificed at each time point described above and lungs were removed, half of it was homogenized and plated on to Middlebrook 7H11 plates in appropriate dilutions to determine CFU; whereas the other half was stored in RNALater (Ambion) for RNA isolation. Spleen was removed and used for *in vitro* restimulation assays. Fig 6A shows a time dependent reduction in CFU counts in lungs of mice treated with G1-4A and subsequently infected with all the three strains, irrespective of their genotype or drug resistance status. Bacterial burden in mouse lung was reduced by 30±11 percent, 48±9 percent and 63±8 percent after 15, 30 and 60 days respectively in case of H37Rv after G1-4A treatment (Fig 6B). For LAM, the bacterial burden was reduced by 26±7 percent, 41±8 percent and 54±6 percent after 15, 30 and 60 days respectively after G1-4A treatment. For Beijing, bacterial burden reduced by 23±10 percent, 44±9 percent and 60±6 percent after 15, 30 and 60 days respectively after G1-4A treatment. In another set of experiments, animals receiving treatment with both INH and G1-4A, exhibited significantly lower levels of bacillary load in their lungs at each time point, compared to the animals receiving only G1-4A or only INH treatment (Fig 6C). Bacterial burden was reduced by 51±14 percent in animals receiving INH +G1-4A as compared to 40±17 and 30±11 percent in animals receiving INH and G1-4A alone respectively, for 15 days (Fig 6D). Further, the percent reduction in bacterial burden was 70±5 percent with INH+G1-4A, 57±6 percent with INH and 48±9 percent with G1-4A treatment, after 30 days, and most remarkably, 89±6 percent with INH+G1-4A, 73±8 percent with INH and 63±8 percent with G1-4A alone, after 60 days of treatment. The time dependent inhibition of bacterial burden in mice lungs treated with INH+G1-4A demonstrates the potential of G1-4A for use in adjunct therapy with current drug regimen.

### Immunomodulation by G1-4A in MTB infected mice

**Fig 7:**
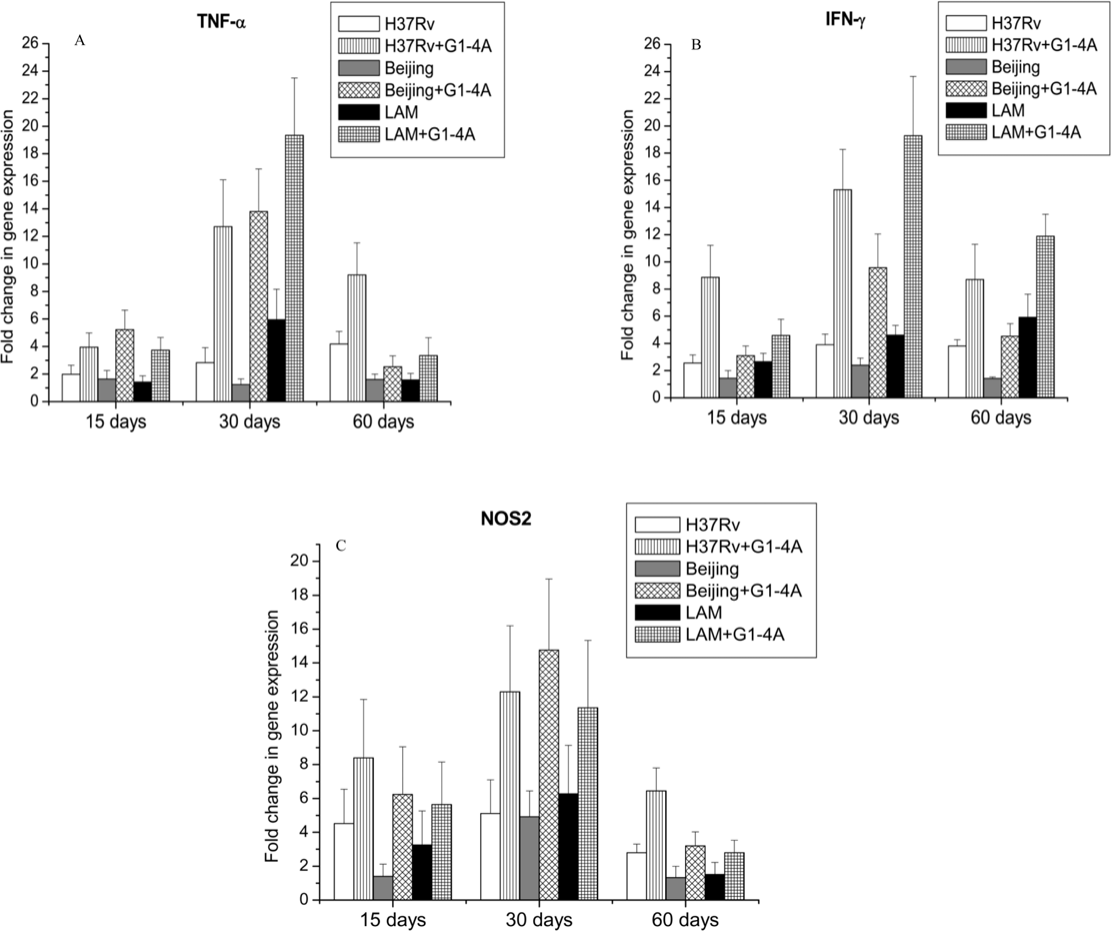
Relative quantitation of gene expression of TNF-α, IFN-γ and NOS2 in lung of MTB infected mice after G1-4A treatment. BALB/c mice were infected with MTB strains and treated with G1-4A. Half of the infected lungs were used to isolate total RNA subjected to real time RT PCR for the quantitative gene expression analysis. (A) TNF-α (B) IFN-γ (C) NOS2. Results shown here are representative of at least three independent experiments performed and presented as mean ± SD.

Further investigations were carried out to elucidate the effect of G1-4A on modulation of immune responses in MTB infected mice. Total RNA from the lung of MTB infected mice was isolated and the expressions of NOS2, TNF-α and IFN-γ were quantitated by real time RT PCR. Fig 7 shows that in presence of G1-4A, expression of TNF-α, IFN-γ and NOS2 is up-regulated in lungs of MTB infected animals. In case of TNF-α, all the three MTB strains induced differential pattern of gene expression in absence of G1-4A but treatment of G1-4A up-regulated the expression of TNF-α after 15 days which peaked at 30 days and declined later on (Fig 7A). Similar pattern was visible in case of IFN-γ (Fig 7B) and NOS2 expression (Fig 7C). Though, G1-4A augmented the expression of above mentioned genes, a marked variation in the fold changes induced by different strains was evident, which could be due to differences in their genotypes or lineage.

**Fig 8:**
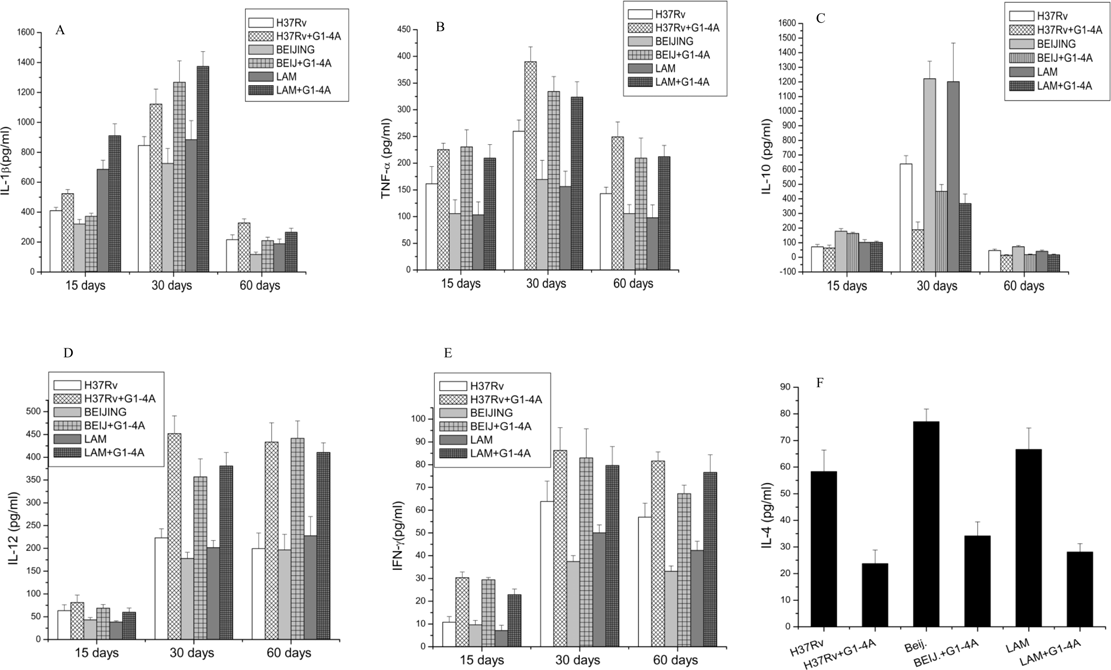
Evaluation of serum cytokines in MTB infected mice after G1-4A treatment. BALB/c mice were infected with MTB strains and treated with G1-4A. Blood was collected from the animals prior to their sacrifice at 15, 30 and 60 days. Serum was used to determine the concentration of given cytokines by ELISA. (A) IL-1β (B) TNF-α (C) IL-10 (D) IL-12(E) IFN-γ (F) IL-4. Results shown here are representative of at least three independent experiments performed and presented as mean ± SD.

Since G1-4A modulated the cytokine responses *in vitro*, we determined the concentration of different cytokines in the serum of infected animals. Blood was collected from the mice by retro-orbital bleeding at 15, 30 and 60 days post treatment, serum was then separated and level of cytokines such as TNF-α, IL-1β, IL-12, IFN-γ, IL-10 and IL-4 was determined by ELISA. Levels of IL-1β and TNF- α (Fig 8A and 8B), in the sera of mice infected with all MTB strains and treated with G1-4A, started increasing at 15 days post infection, peaked to maximum at 30 days and later declined on 60 days post infection. IL-10 levels were highest at 30 days post infection in untreated mice which is a known mechanism by which MTB evades immune system to establish long term infection [10]. Interestingly, however, in animals treated with G1-4A, IL-10 levels fell down substantially, which is beneficial for host (Fig 8C). IL-12 and IFN-γ levels (Fig 8D and 8E) also started increasing at 15 days and remained at the maximum at 30 and 60 days post infection. IL-4 levels were measured at all the time points but it could be detected only at 60 days post infection. IL-4 levels were also higher in all the strains in absence of G1-4A, though a minute variation among the strains was visible but treatment with G1-4A reversed the pattern with decrease in IL-4 levels in animals infected with all strains in presence of G1-4A (Fig 8F). Observations described above, therefore, demonstrate up regulation of Th1 response which is beneficial for the host for the containment and eradication of MTB.

**Fig 9:**
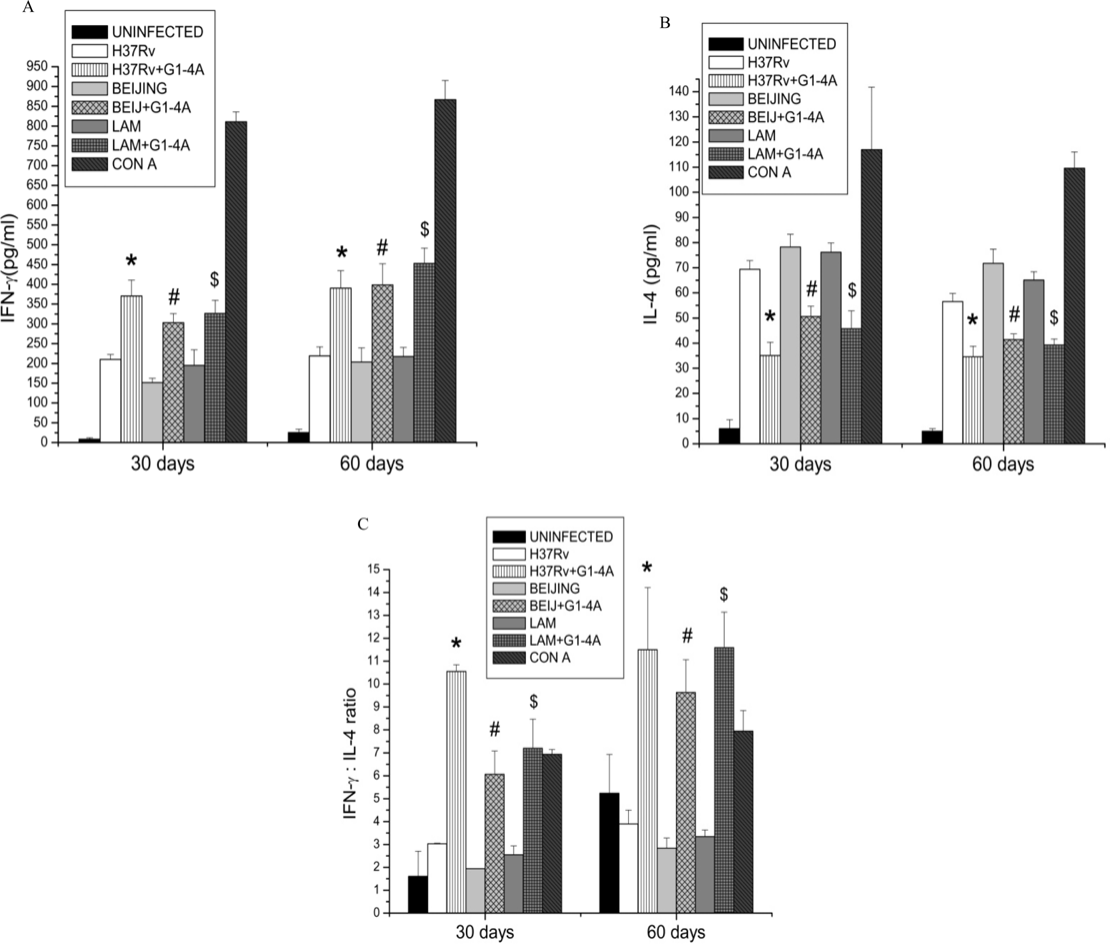
G1-4A enhances IFN-γ and IL-4 ratio in MTB infected mice. Splenocytes isolated from BALB/c after 30 and 60 days of infection either in presence or absence of G1-4A, were treated with PPD and conA for 72 h. Levels of IFN-γ and IL-4 were determined in supernatant. (A) IFN-γ (B) IL-4 (C) IFN-γ/IL-4 ratio. Results shown here are representative of at least three independent experiments performed and presented as mean ± SD.

To further corroborate that G1-4A treatment leads to Th1 stimulation we restimulated splenocytes from infected mice; of both treated and control groups, with PPD and concavalin A (con A) and monitored the level of IFN-γ and IL-4 in supernatants after 72 h by ELISA. It was observed that level of IFN-γ in splenocytes from G1-4A treated animals was significantly higher than that from spleen cells of untreated animals. However, level of IL-4 was lower in case of G1-4A treated animals than untreated animals (Fig 9A and 9B). As Th1 cells produce IFN-γ and Th2 cells produce IL-4 and the ratio of IFN-γ/IL-4 determines the outcome, higher IFN-γ/IL-4 ratio indicates shift towards Th-1 response and lower IFN-γ/IL-4 ratio indicates Th-2 response. IFN-γ/IL-4 ratio in the G1-4A treated animals was significantly greater than that in untreated control mice (Fig 9C). Additionally, to ensure specifically that IFN-γ and IL-4 were produced only by Th cells, splenocytes from each mouse was divided into two groups: one group was incubated with anti-MHC-II (IA^d^) blocking antibodies whereas other group was not incubated with any antibody before restimulation with purified protein derivative of MTB (PPD). IFN-γ and IL-4 were undetectable in the splenocytes pre incubated with anti-IA^d^ blocking antibodies, while control exhibited appropriate IFN-γ and IL-4 response (S2 Fig). Hence it was confirmed that in the present study, IFN-γ and IL-4 were produced by T cells only. Thus our studies establish that in a mouse model G1-4A is effective in the inhibition of intracellular survival of MTB strains irrespective of their drug resistance status and its efficacy may be attributed to up-regulation of NOS2 and Th1 response elicited by G1-4A.

## Discussion

With the advent of multi drug and extremely drug resistant strains of MTB; we are left with very limited options of treatment of tuberculosis. The pace of development of new antibiotics against TB is very slow and any new antibiotic will have its inherent risk of development of drug resistance. In such a scenario targeting the host immune responses looks a promising option as it is largely devoid of the risk of development of drug resistance. Innate immune system is centrally placed at the intersection of microbial detection, inflammation, clearance and cell death, therefore it offers several targets for the therapeutics [13]. Usefulness of several immunomodulators especially TLR agonists have been demonstrated against allergies, cancer and infectious diseases [15]. However, till date, there is no published evidence supporting the effect of any immunomodulator or TLR agonist as a therapeutic agent in MTB infections. We report here for the first time that G1-4A, a polysaccharide immunomodulator and TLR4 agonist from *Tinospora cordifolia*, inhibits the survival of MTB in macrophages as well as in murine infection model.

Polysaccharides isolated from various natural resources affect many biological responses including immune response. Several reports have demonstrated the effects of polysaccharide on various immune regulatory functions such as activation of macrophages, lymphocytes and NK cells, induction of cytokine expression, production of RNI and ROS, antibody production and activation of the complement system [26, 27]. TLRs are one of the largest class of pattern recognition receptors (PRRs) expressed in the cells of innate immune system, that are involved in the detection of invading pathogens by recognizing the characteristic molecular patterns in microorganisms. Apart from microbial components, TLRs display a broad affinity for polysaccharides isolated from various sources of non microbial origin [27]. Macrophages are one of the most important sentinels of innate immune system that play crucial role in host defense and resistance to microbial invasion. Activated macrophages are the key source of ROS, RNI and inflammatory cytokines that are used for defense against microbial invasion [28]. Activated macrophages have been broadly classified in two categories: Classically activated macrophages or M1, and alternatively activated macrophages or M2. In general M1 macrophages possess potent microbicidal properties and are characterized by increased expression of cytokines (TNF-α, IL-1β, IFN-γ, IL-12 and IL-6) enhanced production of NO and up-regulation of MHC-II and CD-86 molecules. Conversely M2 macrophages are characterized by increased expression of IL-4, IL-10 and TGF-β [29] and are poorly microbicidal [30].

Successful pathogens have developed several strategies to circumvent the host immune response for their survival within the host and perturbation of macrophage polarization is one such strategy to undermine the adaptive immunity since macrophages are professional antigen presenting cells [30]. MTB which resides inside macrophages also adapts several strategies to alter macrophage activation. Classically activated macrophages are associated with tuberculostatic and tuberculocidal properties due to production of ROS, NO and other antimicrobial peptides [31]. MTB escapes this hostile environment within the macrophages by creating an environment that supports the macrophage polarization through alternative pathway (M2) resulting in inhibition of NO production, down regulation of TNF-α, IL-6, IL-1β, IL-12 and MHC-II expression, which renders macrophages poorly microbicidal. MTB employs several strategies to achieve this goal, crucial of which is induction of IL-4, TGF-β and IL-10 in macrophages which interferes with IFN-γ signaling and drive the alternative activation of macrophages [32].

Protective responses inhibited by MTB induced IL-10, include blockade of production of pro-inflammatory cytokine TNF-α and Th1 cytokine IL-12, by macrophages and dendritic cells [33, 34]. IL-10 also exerts its inhibitory effect on phagocytosis and bactericidal activities through the inhibition of the production of RNI and ROS by macrophages and down regulation of expression of MHC-II and CD86 having important role in specific immunity [35–37], all of which are key to host immune response to contain or eradicate intracellular pathogen. Another study reported that in absence of IL-10 i.e. in IL-10^-^/^-^ mice, MTB infection resulted into better protection with reduced MTB load in lungs followed by enhanced IFN-γ and Th1 response [38].

Reversal of such inhibitory responses employed by MTB in macrophages may have a promising role in intracellular killing of MTB. Like uninfected macrophages, MTB infected macrophages, when treated with G1-4A, exhibited the up regulation of TNF-α, IL-6, IL-1β, and IL-12 expression, with concomitant increase in NO production by up regulating the NOS2 expression. Additionally, MHC-II and CD86 surface expression was also up regulated in G1-4A treated MTB infected macrophages. G1-4A treatment, also up regulated the phagocytosis of MTB by macrophages. These responses indicate the classical activation of MTB infected macrophages after G1-4A treatment. Consequently, G1-4A treatment inhibited the intracellular survival of MTB H37Rv a drug sensitive strain as well as two clinical isolates with MDR status. Nitric Oxide (NO), one of the inflammatory mediators produced by activated macrophages in response to microbial invasion, contributes to the eradication of microbes including MTB. IFN-γ activated macrophages kill MTB through NO dependent mechanisms [39]. Using L-NAME, a pharmacological inhibitor of NO, we established NO dependent killing of MTB in macrophages treated with G1-4A. Further, we also established that intracellular killing of MTB by G1-4A is TLR4-MyD88 dependent. Uszynski et al have similarly reported that TLR2 dependent NO production in murine macrophages by 19-kDa lipoprotein of MTB was responsible for intracellular killing of MTB [40]. TLR mediated immune responses involve activation of transcription factor NF-κB [41, 42]. Earlier reports have suggested the activation of NF- κB during TLR-MyD88 dependent macrophage activation mediated by plant polysaccharides [27, 43]. Since G1-4A exhibits its effects through TLR4-MyD88 dependent pathway, we hypothesize that G1-4A mediated macrophage activation may involve activation of NF- κB and further study should be undertaken to test this hypothesis.

To assess whether anti-mycobacterial effects of G1-4A would also be seen in an *in vivo* set up; the efficacy of G1-4A was evaluated in a mouse infection model. G1-4A treatment of BALB/c mice inhibited the MTB burden in mice lungs irrespective of genotype or drug resistance status of MTB strains.

Cell mediated immunity (CMI), specifically Th1 response is critical to the control and eradication of mycobacteria in tuberculosis [44]. Th1 cytokines particularly IFN-γ, is key to the control of MTB infection, which activates infected macrophages to initiate the bactericidal response including production of TNF-α and nitric oxide [45]. Up regulation of mRNA expression of IFN-γ, TNF-α and NOS2 was observed in lungs of mice that received G1-4A treatment. The reduction in bacilli count in mice lungs after G1-4A treatment may thus be attributed to increased expression of IFN-γ, TNF-α and NOS2. In addition to this, the levels of cytokines like IFN-γ, TNF-α, IL-1β and IL-12 in the serum of G1-4A treated MTB infected mice were increased. IL-12, another crucial cytokine in controlling MTB, is secreted from antigen presenting cells and drives the Th1 response [45]. Increase in the IL-12 and IFN-γ levels after G1-4A treatment suggests Th1 polarizing potential of G1-4A. Level of IL-10, an anti-inflammatory cytokine, which alters the bactericidal properties of macrophages is increased during MTB infection to enhance the mycobacterial survival. Additionally, MTB is known to manipulate the equilibrium of T cell polarizing cytokines by enhanced expression of IL-4 in infected cells to promote Th2 response which supports the MTB survival [46]. Reduction in the level of IL-10 and IL-4 may have beneficial effects on host and our data demonstrated that G1-4A treatment reduced the serum IL-10 and Il-4 levels in MTB infected mice and this reduction seems to be related with reduced bacterial burden in mice lung after G1-4A treatment.

*In vitro* restimulation of splenocytes from MTB infected mice with PPD led to increase in IFN-γ levels and decrease in IL-4 levels resulting into higher IFNγ/IL-4 ratio which indicates the presence of Th1 polarized cells in G1-4A treated splenocytes. Therefore, it is concluded that G1-4A inhibits the bacterial burden by inducing Th1 response in mice. Further, the enhanced bacterial (H37Rv only) clearance in mice lungs, when G1-4A was given in combination with INH compared to INH alone or G1-4A alone indicated the potential of G1-4A to be used as adjunct to current drug regimen.

In summary our results supports a novel hypothesis that modulation of host immune responses by immunodulators or TLR agonist may have the potential to inhibit the MTB survival both *in vitro* and *in vivo* by activation of macrophages and induction of Th1 response. Our intention here is not to establish G1-4A as therapeutic agent but our data provide a ‘proof of concept’ level demonstration of the above mentioned hypothesis. This clearly shows that G1-4A may offer an attractive option of adjunct therapy for tuberculosis treatment along with current anti-TB drugs. Needless to say, further validation in other models like rabbit, primate and human will be necessary before this promise can be realized.

## Supporting Information

S1 Fig Effect of G1-4A on MTB growth in Middlebrook broth by Resazurin microtiter plate assay (REMA). (DOC)

S2 Fig *In vitro* restimulation assay using anti MHC-II (IA^d^) blocking antibodies to confirm the secretion of IFN-γ and IL-4 by Th cells. (DOC)

S1 Table Primer Sequences. (DOC)
