# Supplementary material for: G1-4A, a Polysaccharide from Tinospora cordifolia Inhibits the Survival of Mycobacterium tuberculosis by Modulating Host Immune Responses in TLR4 Dependent Manner
Source: PLoS One. 2016 May 5;11(5):e0154725. doi: 10.1371/journal.pone.0154725 (PMC4858241; doi:10.1371/journal.pone.0154725)
Supplement: S1 Fig — (DOC) [file pone.0154725.s001.doc]

**Supporting Information**

**S1 Fig. Effect of G1-4A on MTB growth in Middlebrook broth by Resazurin microtiter plate assay (REMA).**

Resazurin microtiter plate assay (REMA) was performed to evaluate the effect of G1-4A on MTB growth in Middlebrook 7H9 broth supplemented with ADC. Briefly, dilutions of G1-4A (62.5µg/ml-1000µg/ml) were prepared in 7H9 broth and dispensed in sterile 96 well plates. Isoniazid was taken as positive control. Inoculums of 10^4^ MTB cells were added to each well. Sterile water was filled to periphery wells to minimize evaporation. The plate was sealed with paraffin, and incubated a 37°C for one week. Resazurin solution (30 µl/well) was added to each well and incubated overnight. A change in color from blue to pink indicated the growth of bacteria. Data shown here are from a single representative experiment out of three independent experiments.
