# Supplementary material for: G1-4A, a Polysaccharide from Tinospora cordifolia Inhibits the Survival of Mycobacterium tuberculosis by Modulating Host Immune Responses in TLR4 Dependent Manner
Source: PLoS One. 2016 May 5;11(5):e0154725. doi: 10.1371/journal.pone.0154725 (PMC4858241; doi:10.1371/journal.pone.0154725)
Supplement: S2 Fig — (DOC) [file pone.0154725.s002.doc]

**Supporting Information**

**S2 Fig. *In vitro* restimulation assay using anti MHC-II (IA^d^) blocking antibodies to confirm the secretion of IFN-γ and IL-4 by Th cells.**

Splenocytes were isolated from the mice infected with H37Rv, infected with H37Rv receiving G1-4A treatment and uninfected. Cells from H37Rv infected mice, either untreated or treated with G1-4A were divided into two groups. Cells of one group were blocked with anti-MHC-II blocking antibodies while cells of other group were not blocked. Later, cells were treated with con A, PPD or left untreated and incubated in humidified environment containing 5% CO2 for 72 h. Levels of IL-4 and IFN-γ were detected by ELISA. (A) IL-4 (B) IFN-γ. Values of at least three independent experiments presented as Mean ± SD).
