# Supplementary material for: G1-4A, a Polysaccharide from Tinospora cordifolia Inhibits the Survival of Mycobacterium tuberculosis by Modulating Host Immune Responses in TLR4 Dependent Manner
Source: PLoS One. 2016 May 5;11(5):e0154725. doi: 10.1371/journal.pone.0154725 (PMC4858241; doi:10.1371/journal.pone.0154725)
Supplement: S1 Table — (DOC) [file pone.0154725.s003.doc]

**Supporting Information**

**S1 Table.** Primer Sequences

| IL-10 (F) | 5’-CGGGAAGACAATAACTG-3’ |
| --- | --- |
| IL-10 (R) | 5’-CATTTCCGATAAGGCTTGG-3’ |
| IL-12 (F) | 5’-CAACATCAAGAGCAGTAGCAG-3’ |
| IL-12 (R) | 5’-TACTCCCAGCTGACCTCCAC-3’ |
| TNF-α (F) | 5’-GGCAGGTCTACTTTGGAGTCATTGC-3’ |
| TNF-α (R) | 5’-ACATTCGAGGCTCCAGTGAATTCGG-3’ |
| IFN-γ (F) | 5’-GGATATCTGGAGGAACTGGC-3’ |
| IFN-γ (R) | 5’-CGACTCCTTTTTCCGCTTCCT-3’ |
| NOS2 (F) | 5’-CCCTTCCGAAGTTTCTGGCAGCAGC3’ |
| NOS2 (R) | 5’-GGCTGTCAGAGCCTCGTGGCTTTGG-3’ |
| GAPDH (F) | 5’-CAAGGCTGTGGGCAAGGTCA-3’ |
| GAPDH (R) | 5’-AGGTGGAAGAGTGGGAGTTGCTG-3’ |
